# Occlusal overload with dental implants: a review

**DOI:** 10.1186/s40729-019-0180-8

**Published:** 2019-07-23

**Authors:** Steven J. Sadowsky

**Affiliations:** Preventive and Restorative Department, University of Pacific Arthur A. Dugoni School of Dentistry, San Francisco, California USA

## Abstract

Controversy persists as to the role of occlusal overload in peri-implantitis. Animal studies have not revealed the biological threshold for fatigue failure in the peri-implant bone. On the other hand, clinical studies have demonstrated a link between parafunction and implant failure, although variables such as intensity and frequency of loads, as well as bone density, have led to different outcomes. The absence of specific engineering “building codes” for the clinician has relegated prosthetic design planning to intuitive guidelines for all patients. For example, higher crown to implant ratios (2–3:1), implant cantilever prostheses and non-splinted restorative designs have been avoided because of the concern for overload. However, evidence has not supported this general approach. A call for preclinical research to establish specific patient load thresholds is in order to establish a customized treatment plan.

## Introduction

According to the American Academy of Implant Dentistry, 500,000 dental implants are placed in the USA annually. These numbers continue to climb despite the unknown role of occlusion in the biological outcomes of osseointegrated implants [1]. Natural teeth that present with initial occlusal discrepancies have demonstrated deeper probing depths and poorer prognoses than those without these discrepancies [2]. However, there are no controlled studies that evaluate the effect of occlusion on implants in humans, due to the fact that they would be countered to the Helsinki accords [3]. What remains is an available body of evidence that is broad and heterogeneous. What is lacking is a study that would reveal the link between specific mechanical loads and histological changes, to serve as a guide to the clinician for implant prosthetic design and occlusal therapy. It is known that the response to increased mechanical stress below a certain threshold will strengthen the bone by increasing the bone density or apposition of the bone [4, 5]. On the other hand, fatigue microdamage resulting in resorption of the bone may be the product of mechanical stress above this threshold [6]. If this gradient could be defined for implant restorations, it would clarify a topic in implant dentistry that has been fueled more by dogma, expert opinion, and inferences from concepts used for natural teeth [7]. The purpose of this study is to review the current preclinical and clinical literature on occlusal overload and its relationship to peri-implant bone loss as well as establish the need for future research.

## Animal studies

Isidor [8–10] published a series of experimental studies using four monkeys to compare the loss of the bone around dental implants following an excessive load or plaque accumulation. He used both machined and titanium dioxide-surfaced implants. After 18 months of loading, the histological result showed that 6 out of 8 loaded implants lost osseointegration. It was concluded that occlusal overload can be the main factor for bone loss for an implant already osseointegrated. In agreement, Miyata et al. [11] experimented with different heights of the superstructure (100, 180, 250 μm) using the same breed of monkeys and found increased bone resorption occurred with an excess of 180 μm or higher after 4 weeks of loading.

However, Heitz-Mayfield et al. [12] using Labrador dogs found no marginal bone loss due to excessive occlusal forces. This study included six dogs and each dog’s bilateral mandibular premolar and molar was removed. After 3 months of healing, two titanium plasma sprayed and two sandblast, large grit acid-etched implants were placed in each side of the mandible in all dogs. A split-mouth design was conducted with supra-occluding crowns on the one side and on the other side unloaded. Plaque control was performed on all implants throughout the experimental time. After 8 months of loading, histology revealed that mineralized bone in contact with the control and test surfaces was not statistically disparate, 72.6% and 73.9%, respectively.

Similar findings were reported by Kozlovsky et al. [13], who performed a split-mouth design on Beagle dogs, placing prosthetic abutments on implants, either in supra-occlusion or infraocclusion. They found no loss of osseointegration or marginal bone loss with non-inflamed, occlusally overloaded prostheses on dental implants. In fact, the authors demonstrated, in the absence of inflammation, there was an increase in the bone to implant contact when overloading the implants.

As is apparent, there are conflicting data in these experimental studies which can be attributed to confounding variables such as differences in experimental design, occlusal overload, and bone quality. Lateral forces applied on the implant prostheses in dogs could only be simulated by artificially created cusp inclines of different types as dogs are unable to perform lateral movements. Some researchers created a lateral displacement on the supra-occluding cusp where others created premature contact in centric occlusion. Finally, different regimens of oral hygiene were used and certainly could not preclude the presence of peri-implant inflammation.

Despite the conflicting evidence, two conclusions have been drawn [7]. Precipitous bone loss from overloading has been shown in a few investigations, but the majority of the more recent animal studies has not replicated these findings. Total loss of osseointegration of an integrated implant appears possible when the applied force exceeds the biological threshold, but this limit is currently unknown, and contingent on the bone quality and possibly the level of inflammation. While it is difficult to quantify the magnitude and direction of naturally occurring occlusal forces, a number of clinical studies may offer clues to appropriate implant/prosthetic treatment planning to minimize peri-implant disease and point to future research.

## Clinical studies

### Bruxism

Twenty percent to 35.9% of patients may generate forces of such magnitude to cause microfracture of the bone around dental implants with concomitant bone loss and implant failure due to bruxism [14–18]. Of note, statistical mediating factors are implant length, diameter, and surface as well as bone quantity D vs. A (Bone quantity relates to the bone volume present. Division A is the height of the bone more than 10 mm. Division B is more than 10 mm in height, but the width at the crest is 2.5–5 mm. Division C is less than 10 mm in height and width atrophied to less than 2.5 mm. Division D is severely deficient bone. Both Division C and D will require augmentation procedures) and bone quality 4 in relation to 1 (Bone quality relates to density present. Type 1 dense bone provides great cortical anchorage but limited vascularity. Type 2 bone is the best bone for osseointegration. Type 3 and 4 bone have soft bone textures with the least success of implant integration in type 4 bone) using Lekholm and Zarb classification [15]. However, given a population of 98 bruxers and matched non-bruxers, considering these variables, the odds ratio of implant failure has been shown to be 2.71 in relation to non-bruxers [16]. Furthermore, Chitumalla et al. [19] in a recent 5-year retrospective study reported a survival rate of dental implants with bruxism habit was 90% after 1 year, 87% after 2 years, 85% after 3 years, 75% after 4 years, and 72% after 5 years. Other investigators have corroborated the link between parafunction and implant failure [20, 21]. This underscores the link between occlusal overload and peri-implant disease and ultimately failure. It is likely that forces applied to implants during bruxism are even higher than those exerted onto natural teeth due to the decreased proprioception of implants. The periodontal ligament of natural teeth provides the central nerve system with feedback for sensory and motor control vs. the implant having feedback from only distant mechanoreceptors and therefore lowers tactile sensitivity (8-fold!) [22]. Another reason is that without a periodontal ligament, implant occlusal loads are directly transferred to the bone leading to higher forces to the supporting structure surrounding implants and risk for bony microfracture (peri-implantitis), compared to natural teeth.

In order to measure bite forces, the most widely accepted recording device is the strain gauge bite force transducer [23–28]. Mean bite force measurements in bruxers have been shown to be 827 N with a standard deviation of 620 N [29]. Bruxers generate both an increased magnitude of force and a higher frequency of tooth contact than non-bruxers [30]. Nishigawa et al. [31] found that 790 N of force was on average generated during bruxism and a mean duration of 7.1 s. Normal masticatory loads have been described as being brief in nature (0.23 to 0.3 contacts/s) at 1 to 2 Hz for a total period of approximately 9 to 17 min per day [32]. Pathologic overloading may also occur as a result of duration of contact. This can magnify loads and stress leading to strain gradients exceeding the physiologic tolerance threshold of the bone, causing microfractures at the bone-implant interface. While repeated single high loads can lead to failure and cause microfractures within the bone tissue, continuous application of low loads may also lead to fatigue failure [33]. The mineralized bone matrix has a mechanical and biologic “memory” for previous stimuli [34].

A microstrain level that is over 4000 is commonly indexed as the threshold for bone fatigue failure [35]. This ceiling varies in accordance with high or low cancellous bone density models with former achieving higher maximums. A high-density cancellous bone (850 Hounsfield units) and a low-density cancellous bone (150 Hounsfield units) would be categorized as type 1 and 4 quality bone, respectively [36, 37]. At the same time, intermittent bouts of 1000 to 3000 microstrains have shown to have a stimulating, anabolic effect on bone mass [38] . This has been explained by Frost [39]. He has identified osteocytes as an important part of the cellular machinery of bone functional adaptation. When the strain stimulus surpasses the homeostatic regulatory mechanism threshold, but is below the bone fatigue failure, tissue level strains lead to fluid flow-mediated osteocyte and dendrite perturbation and release of anabolic factors. In turn, osteoblasts are recruited and the bone is subsequently formed primarily on trabecular and periosteal surfaces—effectively increasing whole bone strength [40].

### Crown to implant ratio

An example of this phenomenon is demonstrated with implants that have a crown to implant ratios of greater than 1 to 1. A number of investigators reported counterintuitive results when high clinical crown to implant ratios (2–3 C/I ratio) did not have the expected catabolic result, but instead caused an anabolic change in the bone [41–44]. The use of short implants when there is a significant interocclusal distance has similarly been successful, even when compared to longer implants with bone augmentation [45–47]. It also offers a methodological pathway to look at relative force/field units in the anabolic/catabolic bone continuum and its tipping point for peri-implantitis.

### Implant cantilever prostheses

Another implant prosthetic design that has been assumed to cause occlusal overload is the cantilever prosthesis. However, systematic reviews and meta-analyses as well as long-term follow-up studies of cantilevers in the partially edentulous patients have demonstrated similar marginal bone levels as the fixed dental prostheses without a cantilever [48–53]. This is irrespective of the use of a mesial or distal cantilever [54]. However, there are limits dictated by the design of the cantilever.

Non-axial forces on natural teeth are mediated through the tensile loading of the principal fibers of the periodontal ligament (PDL), and the occlusal load is transmitted to the surrounding bone [55]. Non-axial loads as in balancing interferences on the teeth have been associated with the significant interproximal bone loss [31]. With implants, the load is transferred directly from the implant to surrounding bone through the ankylosed root analog and adverse effects have not been found to be as pronounced during non-axial loading [56]. However, there are thresholds of non-axial forces that have been shown to cause crestal bone loss around implants. Duyck et al. [57] have shown that a transverse force of 14.7 N applied on a distance of 50 mm from the top of the implant results in a bending moment of 73.5 Ncm when repeated with 2520 cycles at a frequency of 1 Hz causes crater like defects lateral to the osseointegrated implants. This may offer a threshold to explain why fixed dental prostheses that are designed with cantilever arms ≥ 8 mm have demonstrated marginal bone loss, leading to peri-implantitis [58].

### Splinting

The use of splinting to decrease force magnitudes on implant restorations and thereby protect against occlusal overload leading to marginal bone loss continues to be controversial. Vigolo et al. [59] conducted a 10-year split-mouth design on 44 patients with splinted and non-splinted implant restorations on the right and left maxillary posterior quadrant, respectively. He found no difference in crestal bone loss, despite that 17% of the implants were placed in bone quality type IV. However, notable to the patient cohort profile was the absence of any bruxers. Naert et al. [60] treated a larger population with 644 implants. Two hundred thirty-five were restored with single crowns and 409 with a splinted prosthesis. After a 16-year follow-up, 95.8% of all restorations survived. Statistical analysis showed no significant difference in hazard rate between implant-supported single crowns and those splinted by means of fixed prostheses. It was also shown that neither restoration design, jaw site nor implant position (anterior or posterior) had a significant effect on bone loss [61]. Despite the fact that patients were widely accepted for treatment, no data were reported on the number of bruxers. While recommendations have been made to splint crowns in patients with parafunction [62, 63], no clinical or histological evidence has demonstrated marginal bone response advantages.

## Discussion

Treatment planning implant-prosthetic rehabilitation should be dependent on a biomechanical algorithm customized for each patient. Given the lack of a definitive load-bearing analysis for bone supporting implants, an empirical or intuitive dogma, based on a tooth model, has proliferated in the clinical amphitheater. This has led to a penchant for invasive and costly procedures rather than more minimally invasive approaches. While short implants (even with high crown to implant ratios) can successfully override augmentation procedures, and implant cantilevers can dependably reduce surgical exposure as well as soft tissue problems, and splinting may not be necessary to maintain the marginal bone level; biases against short implants, implant cantilevers, and non-splinted units are replete and are not evidence-based. Despite this so-called safe approach to implant treatment planning, peri-implantitis is on the rise. Understanding the role that mechanical stress and strain play in peri-implant bone loss may change the tooth model as a guide for implant prosthetic designs and assist in explaining why peri-implantitis has been so prevalent.

This study is limited by the non-systematic nature of its review. However, there are no randomized or prospective human trials on occlusal overload due to the ethical concerns in such methodologies. The way forward will be in designing and conducting overload conditions in preclinical experiments and using finite element analyses to identify thresholds of bone microstrain for assessing safe implant/prosthetic designs for our patients.

## Summary

When the best available evidence is lacking in directing clinicians in their treatment planning decisions with implant restorations, a basic science approach can offer clarity. The relationship between mechanical loading and biologic consequences on bone response has been established, but specific thresholds have not been correlated to prosthetic design and occlusal scheme guidelines. The high ceiling of implant survival has clouded the importance of dissecting why some implants fail. It has been sobering, however, to note that as high as 20% of all implant patients experience peri-implantitis, while the impact of occlusal overload remains unknown.

## Data Availability

N/A.
